# Augustus Philemon Head, M. D.

**Published:** 1913-08

**Authors:** 


Augustus Philemon Head, M. D., Rush, 1884, one of the general editors of the Practical Medicine Series of Year Books, died of pneumonia in Austin, Chicago, Ill., June 11, at the age of 51.

